# Energy-Oriented Hybrid Cooperative Adaptive Cruise Control for Fuel Cell Electric Vehicle Platoons

**DOI:** 10.3390/s24155065

**Published:** 2024-08-05

**Authors:** Shibo Li, Liang Chu, Pengyu Fu, Shilin Pu, Yilin Wang, Jinwei Li, Zhiqi Guo

**Affiliations:** 1 College of Automotive Engineering, Jilin University, Changchun 130022, China; lisb22@mails.jlu.edu.cn (S.L.); chuliang@jlu.edu.cn (L.C.); wyl22@mails.jlu.edu.cn (Y.W.); lijw22@mails.jlu.edu.cn (J.L.); 2 National Key Laboratory of Automotive Chassis Integration and Bionics, Jilin University, Changchun 130022, China; 3 Department of Aeronautical and Automotive Engineering, Loughborough University, Loughborough LE11 3TU, UK; p.fu@lboro.ac.uk; 4 GAC R&D Center, Guangzhou 511434, China; pushilin@gacrnd.com

**Keywords:** energy-oriented hybrid cooperative adaptive cruise control (eHCACC), fuel cell electric vehicle platoon, energy management strategy (EMS)

## Abstract

Given the complex powertrain of fuel cell electric vehicles (FCEVs) and diversified vehicle platooning synergy constraints, a control strategy that simultaneously considers inter-vehicle synergy control and energy economy is one of the key technologies to improve transportation efficiency and release the energy-saving potential of platooning vehicles. In this paper, an energy-oriented hybrid cooperative adaptive cruise control (eHCACC) strategy is proposed for an FCEV platoon, aiming to enhance energy-saving potential while ensuring stable car-following performance. The eHCACC employs a hybrid cooperative control architecture, consisting of a top-level centralized controller (TCC) and bottom-level distributed controllers (BDCs). The TCC integrates an eco-driving CACC (eCACC) strategy based on the minimum principle and random forest, which generates optimal reference velocity datasets by aligning the comprehensive control objectives of the platoon and addressing the car-following performance and economic efficiency of the platoon. Concurrently, to further unleash energy-saving potential, the BDCs utilize the equivalent consumption minimization strategy (ECMS) to determine optimal powertrain control inputs by combining the reference datasets with detailed optimization information and system states of the powertrain components. A series of simulation evaluations highlight the improved car-following stability and energy efficiency of the FCEV platoon.

## 1. Introduction

With the rapid growth in travel demand, issues that hinder emissions reduction, fuel efficiency, congestion reduction, and safety improvements have significantly increased, drawing attention to the development of sustainable transportation systems [1,2]. Fuel cell electric vehicles (FCEVs) are widely recognized for their environmental friendliness and the broad substitutability of hydrogen energy [3], increasingly considered an effective tool for sustainable transportation. Although FCEVs exhibit impressive emissions and range performance, their power components display complex non-linear characteristics and poor dynamic response [4,5], making it challenging to fully utilize their energy-saving potential. Additionally, with the rapid development of intelligent transportation systems (ITS) [6] and vehicle-to-everything (V2X) technology [7], FCEVs in platoons can achieve significant sustainability improvements through information sharing and coordinated optimization [8]. However, co-optimization with the simultaneous consideration of following performance and the intricacies of energy-saving and inter-vehicle coordinated control limit the economic, safety, and stability improvements [9,10,11]. Therefore, it is urgent to develop an energy-efficient inter-vehicle coordinated control method for FCEV platoons.

Energy management strategies (EMSs) for FCEVs are generally classified into three categories: rule-based, learning-based strategies, and optimization-based [12]. Rule-based EMS, such as state machine control [13], are developed based on expert experience. While rule-based EMS offer excellent real-time performance and stability [14], their fixed rules limit adaptability and effectiveness in diverse driving conditions, restricting the collaborative energy-saving potential of FCEV platoons. Learning-based EMS, such as reinforcement learning methods [15], dynamically optimize control sequences through numerous iterations, ensuring optimal control performance in specific driving scenarios. However, these methods require extensive data preparation, detailed hyperparameter tuning, and offline training under predefined driving conditions [16], posing challenges for practical applications aimed at improving economic performance. Optimization-based EMS can be divided into global optimization EMS and instantaneous optimization EMS [17]. Representative methods of global EMS include dynamic programming (DP) [18] and Pontryagin’s minimum principle (PMP) [19], which obtain the optimal control sequence by traversing all control variables based on pre-acquired global driving information. The high computational resource requirements and unknown future driving information hinder real-time applications in energy management [20]. As an instantaneous EMS, model predictive control (MPC) [21] captures system states within a specific predictive horizon for rolling optimization to solve for the optimal control sequence. While MPC balances global control performance and real-time requirements through short-term future state predictions, even though generating future reference datasets can improve control performance, the generation of highly accurate datasets is challenging [22], and the accuracy of state observations significantly impacts control performance [23]. Another primary instantaneous approach is the equivalent consumption minimization strategy (ECMS) [24], which introduces equivalent factors to convert electrical energy consumption into fuel consumption, solving for the optimal control input at a given transient state. This approach significantly reduces dependence on computational resources [25], making it more suitable for scenarios involving multiple electric vehicles. Therefore, ECMS is considered a superior solution for tapping into the energy-saving potential of fuel cell vehicle powertrain components. Even so, the effectiveness of energy management strategies in scenarios involving multiple FCEV platoons is influenced by inter-vehicle coordinated control [26,27]. Consequently, a strategy that simultaneously considers inter-vehicle coordinated control and energy management is essential for fully realizing the energy-saving potential of FCEV platoons while maximizing car-following performance, safety, and stability.

To balance vehicle coordination and energy economy, the interaction between following and leading vehicles in a dual-vehicle scenario has been widely studied [28]. For mixed traffic scenarios involving electric vehicles, connected vehicles, and human-driven vehicles, a series of eco-driving adaptive cruise control (Eco-ACC) systems have been proposed [29]. Eco-ACC has achieved excellent inter-vehicle coordination and energy-saving control [30]. However, large velocity and acceleration fluctuations of leading vehicles can cause stability loss and reduced energy efficiency for following vehicles in a platoon of three or more electric vehicles. Cooperative adaptive cruise control (CACC) [31] for multiple electric vehicles is considered an effective solution to this problem. However, the integration of energy-saving controls and existing inter-vehicle coordination including car-following errors [32], comfort [33], stability [34], topology [35], and communication failures [36] is complex, increasing difficulty for the economic coordination of the platoon. Recently, advanced eco-CACC [37] has emerged to address the issue of economic driving inter-vehicle coordination in multi-vehicle scenarios. The mainstream implementation of eco-CACC is velocity planning [38], aiming at optimizing platoon velocity by integrating road network information from ITS [39], inter-vehicle communication [40], and aerodynamic theory [41] to reduce energy consumption under complex constraints. In eco-CACC, velocity planning methods mainly include rule-based, optimization-based, and reinforcement learning-based approaches [42]. Rule-based methods adjust velocity through predefined rules [43,44], achieving simplicity and fast computation but poor adaptability, resulting in unsuitable for complex and diverse traffic conditions. Optimization-based methods, such as trajectory optimization for connected autonomous vehicles at signalized intersections [45] and hierarchical reinforcement learning-based eco-driving optimization [46], use dynamic programming algorithms to find optimal velocity sequences within given time and space constraints, but have high computational complexity and poor real-time performance. Reinforcement learning-based methods, such as multi-agent deep reinforcement learning for eco-driving [47] and cooperative multi-agent reinforcement learning for adaptive cruise control [48], offer superior adaptability and can handle complex traffic scenarios, but have complex and time-consuming training processes. Furthermore, even if the reference velocity dataset generated from velocity planning is accurate enough, the eco-CACC control architecture still affects the effectiveness and efficiency of coordinated control [49]. Consequently, a customized cooperative framework combining high-precision velocity planning and energy management strategies is needed for economic driving coordination. To sum up, detailed literature reviews emphasize the urgent need to develop a superior eco-CACC strategy that integrates precise inter-vehicle coordinated control and energy management, ensuring the safety, stability, and economy of FCEV platoons.

In light of the discussion, an energy-oriented hybrid cooperative adaptive cruise control (eHCACC) strategy is proposed for an FCEV platoon, targeting improving energy-saving potential and ensuring stable car-following performance. In eHCACC, a hybrid cooperative control architecture is employed, including a top-level centralized controller (TCC) and bottom-level distributed controllers (BDCs). The TCC of the proposed eHCACC integrates an eco-driving CACC (eCACC) strategy based on the minimum principle and random forest, which achieves the generation of optimal reference velocity datasets by integrating the comprehensive control objectives of the platoon during longitudinal following, thereby accommodating both the car-following performance and the economy in the perspective of the vehicle platoon. In the BDCs, the ECMS solves for the optimal control inputs to the powertrain by combining the generated reference datasets with comprehensive optimization information and the operating states of the powertrain components, achieving a complete release of energy-saving potential from the perspective of the individual vehicle. The contributions are illustrated in:

1. An energy-oriented hybrid cooperative adaptive cruise control strategy is proposed for an FCEV platoon. This hybrid control architecture combines a novel eco-driving CACC with superior energy management, harmonizing the car-following performance and economy of the platoon.
2. The eco-driving CACC strategy generates optimal reference velocity datasets by integrating the comprehensive control objectives during longitudinal following of the platoon, including spacing error, speed error, and economy error, thus realizing the co-optimization of the following error and the energy efficiency.
3. By parsing the comprehensive reference information, the superior energy management based on ECMS realizes the rational control of multiple energy sources of individual vehicles, thus completely releasing the energy-saving potential of the vehicle platoon.

The remainder of this paper is organized as expressed. Section 2 introduces the modeling of an FCEV platoon. The eHCACC strategy is proposed in Section 3. The simulation and discussion are provided in Section 4. Finally, conclusions are presented in Section 5.

## 2. System Modeling

**Figure 1:**
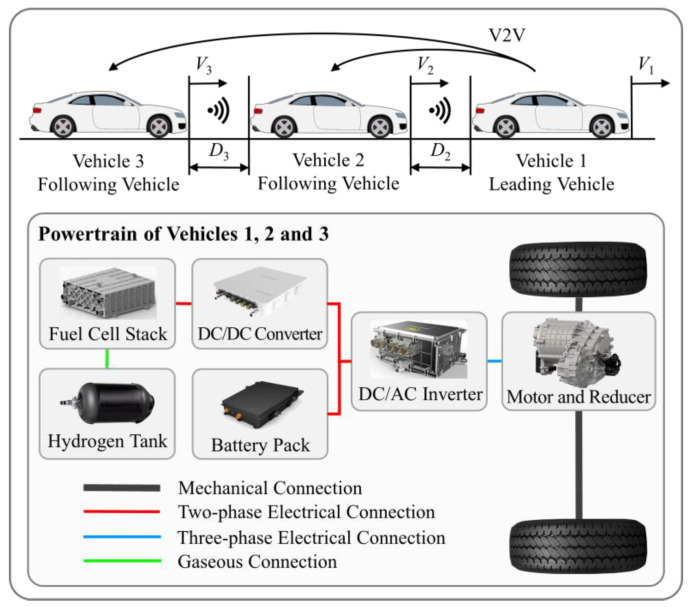
Homogeneous vehicle platoon consisting of FCEVs under the PLF communication topology.

Consider a homogeneous vehicle platoon consisting of three fuel cell electric vehicles (FCEVs) as shown in Figure 1, where the leading vehicle is labeled 1 and the following vehicles are named 2 to 3. Moreover, due to the superior performance of anti-interference, the vehicle platoon adopts the predecessor–leader–following (PLF) communication topology [50]. The displacement, velocity, and acceleration information of neighboring or leading vehicle is obtained by using on-board sensors, radars and vehicle-to-vehicle (V2V), to plan movement of following vehicles at the next moment. To ensure the effectiveness and consistency of economic performance verification, the velocity of leading vehicle is determined by a standard driving cycle. Moreover, in each individual, the studied FCEV consists of a fuel cell system, a battery pack, a motor and its reducer, a unidirectional DC/DC converter, and a bidirectional DC/AC converter. The fuel cell system and battery pack provide energy to the motor through DC/AC converter, thereby driving the vehicle. The detailed parameters are shown in Table 1.

### 2.1. Vehicle Platoon Modeling

#### 2.1.1. Spacing Policy

Considering the motion information of leading vehicle, the spacing policy based on constant time headway (CTH) is employed. Hence, the reference position of following vehicles can be expressed as:

(1) $$\left\{\begin{array}{l} S_{ref,i}(t)=S_{1}(t)-D_{ref,i}(t)\\ D_{ref,i}(t)=h\cdot V_{i}(t)+L \end{array}\right.$$

where $S_{ref,i}$, $D_{ref,i}$, and $V_{i}$ are the reference displacement, reference spacing, and velocity of following vehicle i, respectively. $S_{1}$ is the displacement of leading vehicle. L is the fixed reference spacing, and h is the time headway.

#### 2.1.2. Longitudinal Dynamics Model

For the studied vehicle platoon, lateral motion, vertical motion, and tire slip are ignored. Hence, the nonlinear longitudinal dynamics model of the vehicle platoon is written as:

(2) $$\left\{\begin{array}{l} {\dot{S}}_{i}(t)=V_{i}(t)\\ {\dot{V}}_{i}(t)=\frac{1}{m_{i}}\left(\frac{T_{i}(t)}{\eta_{i}\cdot r_{i}}-m_{i}gf_{i}-\frac{C_{D,i}A_{i}V_{i}^{2}(t)}{21.15}\right) \end{array}\right.$$

where S and V represent as the displacement and velocity, respectively. T is the actual torque of vehicle, η is the transmission system efficiency, r is the tire rolling radius, m is the vehicle mass, g is the gravitational acceleration, f is the rolling resistance coefficient, $C_{D}$ is the aerodynamic drag coefficient, and A is the vehicle frontal area. The subscript i represents the vehicle i.

Considering the inertia hysteresis of vehicles, the actual vehicle torque can be expressed as:

(3) $${\dot{T}}_{i}(t)=(T_{Motor,i}(t)\cdot i_{i}-T_{i}(t))/\tau_{i}$$

where $T_{Motor,i}$ is the motor torque, $i_{i}$ is the transmission ratio, and $\tau_{i}$ is the time-delay constant of the longitudinal dynamical system.

### 2.2. Powertrain Modeling

In the homogeneous vehicle platoon, FCEV individuals have the same powertrain. The powertrain demand power can be expressed as:

(4) $$\begin{array}{l} P_{load}(t)=\gamma_{Motor}(t)\cdot P_{Motor}(t)\\ \gamma_{Motor}=\left\{\begin{array}{cc} 1/\eta_{Motor}(t) & ,\mathrm{Driving}\\ \eta_{Motor}(t) & ,\mathrm{Braking} \end{array}\right. \end{array}$$

where $P_{load}$ is the powertrain demand power. $\gamma_{Motor}$ is the efficiency factor. $P_{Motor}$ and $\eta_{Motor}$ are mechanical power and efficiency of the motor, respectively.

When driving the vehicle, the powertrain demand power is provided by the fuel cell system and the battery, and their coordination relationship is determined by an energy management strategy, therefore, using the following equation:

(5) $$\left\{\begin{array}{l} P_{FC}(t)=u_{ES}(t)\cdot P_{load}(t)\\ P_{Batt}(t)=\left(1-u_{ES}(t)\right)\cdot P_{load}(t) \end{array}\right.$$

where $P_{FC}$ and $P_{Batt}$ are output power of the fuel cell and the battery, respectively. $u_{ES}$ is the power distribution coefficient between the fuel cell system and the battery.

#### 2.2.1. Fuel Cell Model

**Figure 2:**
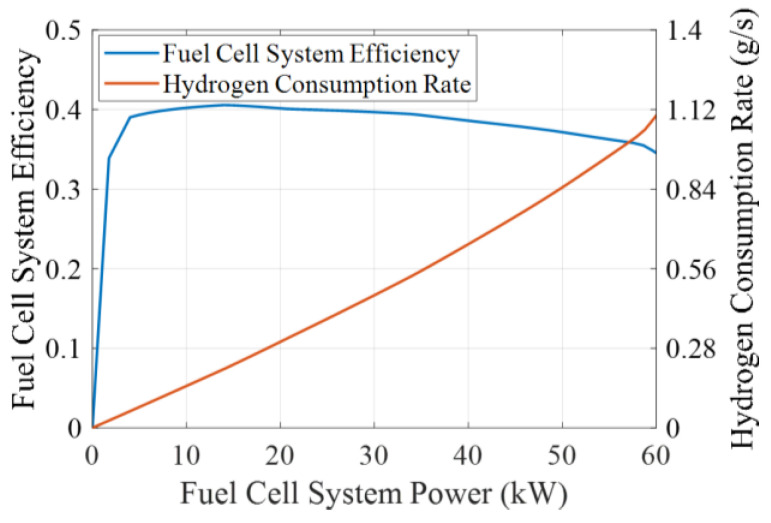
Efficiency and hydrogen consumption characteristics of the fuel cell system.

One proton exchange membrane fuel cell (PEMFC) is employed for each FCEV individual. Since the focus of this study is on the car-following performance and economy of the vehicle platoon, the influence of external factors such as gas pressure and ambient temperature on the performance of the fuel cell system is ignored. Therefore, look-up tables are used to reflect the efficiency and energy consumption characteristics of the fuel cell system, as shown in Figure 2.

Theoretically, the hydrogen consumption of the fuel cell system can be expressed as its relation to power and efficiency:

(6) $$m_{H_{2}}(t)=\frac{1}{LHV_{H_{2}}}\int \frac{P_{FC}(t)}{\eta_{FC}(P_{FC}(t))}dt$$

where $m_{H_{2}}$ is the hydrogen consumption of the fuel cell system, $LHV_{H_{2}}$ is the hydrogen low calorific value, here taken as 120 MJ/kg. $\eta_{FC}$ is the fuel cell system efficiency, which is a function of output power of the fuel cell system.

After simplification, the hydrogen consumption of the fuel cell system is expressed as a relationship with the hydrogen consumption rate as given below:

(7) $$m_{H_{2}}(t)=\int P_{FC}(t)\cdot C_{H_{2}}(P_{FC}(t))dt$$

where $C_{H_{2}}$ is the hydrogen consumption rate shown in Figure 2, which is also a function of output power of the fuel cell system.

#### 2.2.2. Battery Model

**Figure 3:**
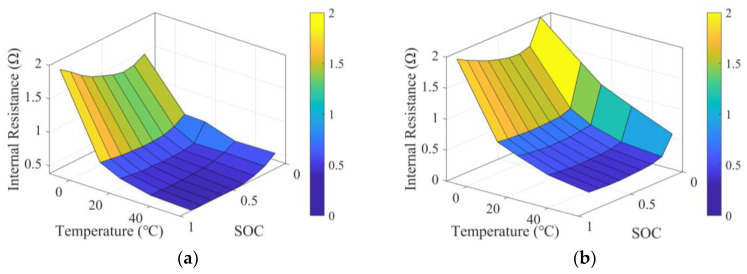
Internal resistance characteristics of the battery: (**a**) charging internal resistance; (**b**) discharging internal resistance.

The battery mounted on each FCEV individual is modeled as a first-order resistance-capacitance (RC) model, ignoring electrochemical characteristics. Therefore, the dynamic characteristics of the battery are written as:

(8) $$\left\{\begin{array}{l} U_{Batt}(t)=U_{BattOCV}-R_{Batt}(t)I_{Batt}(t)\\ I_{Batt}(t)=\frac{U_{BattOCV}-\sqrt{U_{BattOCV}^{2}-4R_{Batt}(t)P_{Batt}(t)}}{2R_{Batt}(t)}\\ R_{Batt}(t)=f(Temp,SOC(t))\\ SOC(t)=SOC(t_{0})-\frac{\int_{t_{0}}^{t_{end}}I_{Batt}(t)dt}{3600C_{Batt}} \end{array}\right.$$

where $U_{Batt}$ is the battery terminal voltage, $U_{BattOCV}$ is the battery open circuit voltage, and $I_{Batt}$ is the battery current. $R_{Batt}$ is the battery internal resistance, which is a function of battery temperature and battery SOC, as shown in Figure 3. SOC is the battery SOC, and $C_{Batt}$ is the battery capacity.

Due to the power loss of the battery internal resistance, the battery power and power loss are expressed as:

(9) $$\left\{\begin{array}{l} P_{Batt}(t)=U_{Batt}(t)I_{Batt}(t)\\ P_{BattLoss}(t)=I_{Batt}^{2}(t)R_{Batt}(t) \end{array}\right.$$

Battery efficiency is defined by the charging and discharging processes of the battery and is expressed as:

(10) $$\eta_{Batt}(t)=\left\{\begin{array}{c} \frac{P_{Batt}(t)}{P_{Batt}(t)+P_{BattLoss}(t)},P_{Batt}(t)>0\\ \;\;\;\;1\;\;\;\;,\;P_{Batt}(t)=0\\ \frac{P_{Batt}(t)+P_{BattLoss}(t)}{P_{Batt}(t)},P_{Batt}(t)<0 \end{array}\right.$$

where $\eta_{Batt}$ is the battery efficiency, and $P_{Batt}$ and $P_{BattLoss}$ are power and its loss of the battery, respectively.

#### 2.2.3. Motor Model

**Figure 4:**
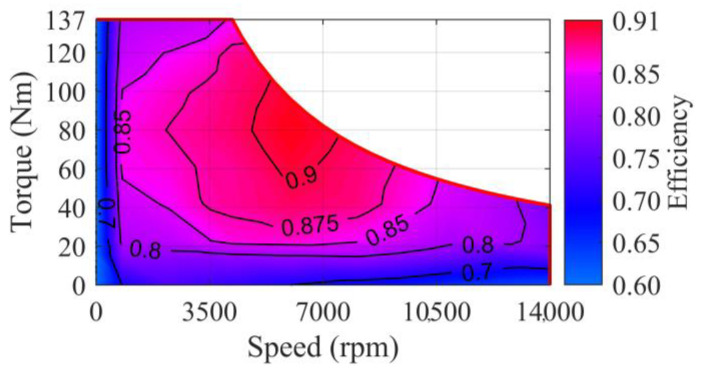
Efficiency characteristics of the motor.

Owing to the rapid torque response of the motor, the powertrain inertial lags are not considered in this paper. Hence, the motor efficiency is written as a function of its torque and speed:

(11) $$\eta_{Motor}(t)=f(T_{Motor}(t),n_{Motor}(t))$$

where $\eta_{Motor}$, $T_{Motor}$, and $n_{Motor}$ are the efficiency, torque, and speed of the motor, respectively. The mapping relationship is shown in Figure 4.

Meanwhile, the motor mechanical power is expressed as:

(12) $$P_{Motor}(t)=\frac{T_{Motor}(t)\cdot n_{Motor}(t)}{9550}$$

where $P_{Motor}$ represents the motor mechanical power.

## 3. Energy-Oriented Hybrid CACC 

**Figure 5:**
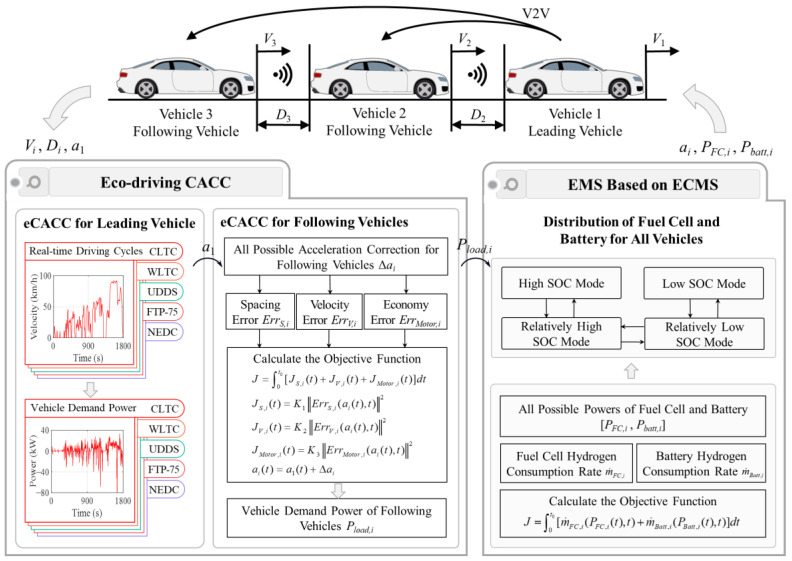
Energy-oriented hybrid CACC architecture.

In order to maximize the energy-saving potential of the FCEV platoon while ensuring stable car-following, an energy-oriented hybrid cooperative adaptive cruise control (eHCACC) method tailored for the FCEV platoon is proposed. This hybrid cooperative control architecture consists of a top-level centralized controller and bottom-level distributed controllers, as shown in Figure 5.

In the top-level centralized controller, a novel car-following method considering economic optimization is proposed for the FCEV platoon, aiming at improving following stability and economy in longitudinal dynamics. Specifically, eco-driving CACC (eCACC) implements the generation of optimal reference datasets based on minimum principle and random forest by radar signal acquisition and processing, comprehensively considering control objectives during the longitudinal following process, which enables the provision of accurate reference sequences for the bottom-level distributed controllers to release the energy-saving potential with the guarantee of the car-following performance.

In the bottom-level distributed controller, energy management strategy (EMS) based on equivalent consumption minimization strategy (ECMS) is proposed to further improve energy-saving performance. To be specific, by receiving reference sequences with comprehensive optimization information sent by the top-level centralized controller, the ECMS solves the optimal control sequences of the powertrain by combining the operating states of the system components, aiming at precisely realizing the control objectives of optimal following and the economic performance of the vehicle platoon.

### 3.1. Minimum Principle for eCACC and EMS

As a method for solving optimal control problems under a nonlinear system of ordinary differential equations, Pontryagin’s minimum principle (PMP) [19] allows the solving of the optimal control inputs to minimize the objective function and Hamiltonian function under given constraints. The mathematical equation of a control system can be expressed as:

(13) $$\dot{x}(t)=f(x(t),u(t),t)$$

where x is the state variables and u is the control inputs.

Furthermore, the objective function of the control system can be written as a combination of end states and transition costs:

(14) $$J=h(x(t_{end}))+\int_{t_{0}}^{t_{end}}L(x(t),u(t),t)dt$$

where $h(x(t_{end}))$ is the end states, and $\int_{t_{0}}^{t_{end}}L(x(t),u(t),t)dt$ is the transition costs. 

In order to minimize the objective function, the corresponding Hamiltonian function can be expressed as:

(15) $$H(x(t),u(t),\lambda (t),t)=L(x(t),u(t),t)+\lambda^{T}(t)f(x(t),u(t),t)$$

where $\lambda^{T}(t)$ is the Lagrange multiplier vector. For the optimal control inputs $u^{*}$, the state variables and Lagrange multiplier vector are required to satisfy the following regular equations and boundary conditions:

(16) $$\left\{\begin{array}{l} \dot{x}(t)={\left.\frac{\delta H}{\delta \lambda }\right|}_{u^{*}(t)}=f(x^{*}(t),u^{*}(t),t)\\ {\dot{\lambda }}^{*}(t)={\left.-\frac{\delta H}{\delta x}\right|}_{u^{*}(t)}=-\frac{\delta L}{\delta \lambda }(x^{*}(t),u^{*}(t),t)-\lambda^{*}(t){\left[\frac{\delta f}{\delta \lambda }(x^{*}(t),u^{*}(t),t)\right]}^{T}\\ x^{*}(t_{0})=x_{0}\\ x^{*}(t_{end})=x_{end} \end{array}\right.$$

where $x_{0}$ and $x_{end}$ are the initial and end states of the control system.

Therefore, under the above constraints, the control value that satisfies the Hamiltonian function in a finite set achieving the minimum is the optimal control input, which is expressed as:

(17) $$u^{*}(t)=\arg\underset{u(t)}{\min}[H(x^{*}(t),u(t),\lambda (t),t)]$$

Therefore, the minimum principle can solve multi-objective optimization problems under diverse constraints. In the optimal control problem of energy-oriented CACC, the control objectives and constraints are fraught with diversity, thus affecting the optimal control effectiveness.

### 3.2. Eco-Driving CACC in Top-Level Centralized Controller

**Figure 6:**
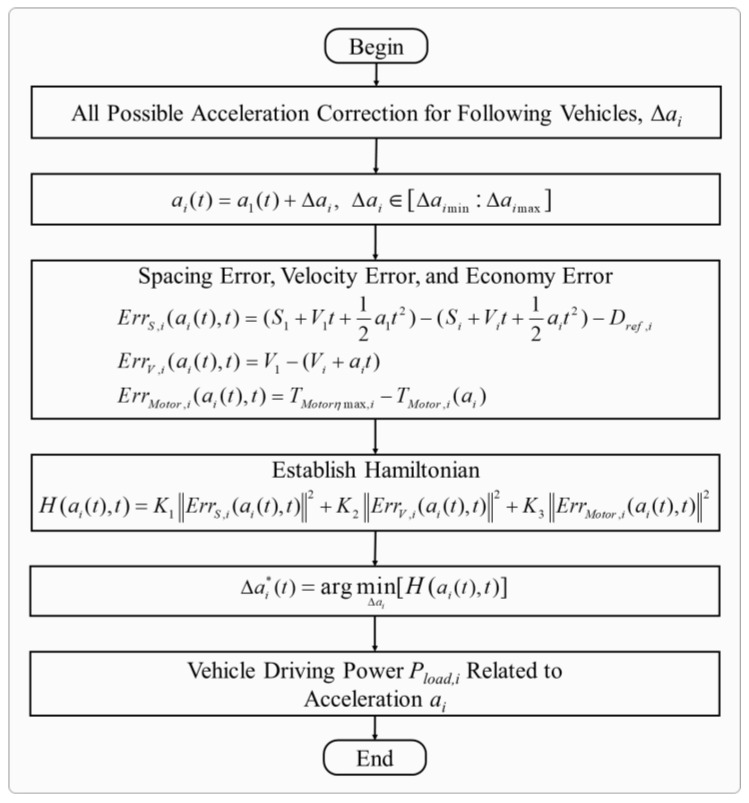
Implementation of eco-driving CACC.

eCACC is proposed for the FCEV platoon, designed to maximize the synergistic economy of the vehicle platoon while ensuring car-following performance. Moreover, eCACC provides reference datasets with comprehensive optimization information for the bottom-level distributed controllers to further improve the energy efficiency of powertrain components. The implementation of eco-driving CACC is shown in Figure 6.

To be specific, in the control architecture of eCACC, the system variables for vehicle i in the vehicle platoon at moment k are defined as follows:

(18) $$\left\{\begin{array}{l} x_{i}(k)={\left[S_{i}(k),V_{i}(k),a_{i}(k)\right]}^{T}\\ y_{i}(k)=[Err_{S,i}(k),Err_{V,i}(k),Err_{Motor,i}(k)]\\ u_{i}(k)=\Delta a_{i}(k) \end{array}\right.$$

where x, y, and u represent the state variables, measurements, and control inputs, respectively. S, V, and a are the displacement, velocity, and acceleration, respectively. $Err_{S}$, $Err_{V}$, and $Err_{Motor}$ are the spacing error, velocity error, and economy error, respectively. $\Delta a_{i}$ denotes the acceleration correction.

The discrete longitudinal dynamics model of following vehicles in the platoon can be represented by the following state-space equation:

(19) $$\begin{array}{l} x_{i}(k+1)=A_{1}x_{i}(k)+B_{1}x_{1}(k)+C_{1}u_{i}(k)\\ \left\{\begin{array}{l} A_{1}=\left[\begin{array}{ccc} 0 & T_{S} & T_{S}^{2}/2\\ 0 & 1 & T_{S}\\ 0 & 0 & 1-T_{S}/\tau_{i} \end{array}\right]\\ B_{1}=\left[\begin{array}{ccc} 0 & \cdots & 0\\ \vdots & \ddots & \vdots \\ 0 & \cdots & T_{S}/\tau_{i} \end{array}\right]\\ C_{1}={\left[0,0,T_{S}/\tau_{i}\right]}^{T} \end{array}\right. \end{array}$$

where $T_{S}$ denotes the sampling time. $A_{1}$, $B_{1}$, and $C_{1}$ are coefficient matrices.

Under the CTH spacing policy, several critical errors are defined to improve economy of the vehicle platoon while ensuring the car-following performance, which are expressed as:

(20) $$\left\{\begin{array}{l} Err_{S,i}(k)=S_{1}(k)+\Delta S_{1}(k)-S_{i}(k)-\Delta S_{i}(k)-D_{ref,i}(k)\\ Err_{V,i}(k)=V_{1}(k)-V_{i}(k)-\Delta V_{i}(k)\\ Err_{Motor,i}(k)=T_{Motor\eta \max,i}-m_{i}r_{i}/i_{i}\cdot (a_{1}(k)+\Delta a_{i}(k)) \end{array}\right.$$

where $\Delta S_{1}$ and $\Delta S_{i}$ are represent the displacement correction of vehicles 1 and i, respectively. $\Delta V_{1}$ and $\Delta V_{i}$ are the velocity correction of vehicle 1 and i, respectively. $T_{Motor\eta \max,i}$ denotes the motor torque of vehicle i corresponding to the maximum efficiency at the current motor speed. Note that following vehicles enable access to the operational status of the leading vehicle through vehicle-to-vehicle communication. 

Hence, the observation equation can be expressed as:

(21) $$\begin{array}{l} y_{i}(k)=A_{2}x_{i}(k)+B_{2}x_{1}(k)+C_{2}u_{i}(k)+D_{2}\\ \left\{\begin{array}{l} A_{2}=\left[\begin{array}{ccc} -1 & -T_{S}-h & 0\\ 0 & -1 & 0\\ 0 & 0 & 0 \end{array}\right]\\ B_{2}=\left[\begin{array}{ccc} 1 & T_{S} & 0\\ 0 & 1 & -T_{S}\\ 0 & 0 & -m_{i}r_{i}/i_{i} \end{array}\right]\\ C_{2}=-{\left[T_{S}^{2}/2,T_{S},m_{i}r_{i}/i_{i}\right]}^{T}\\ D_{2}={\left[-L,0,T_{Motor\eta \max,i}\right]}^{T} \end{array}\right. \end{array}$$

where $A_{2}$, $B_{2}$, $C_{2}$, and $D_{2}$ are coefficient matrices.

In order to prevent the acquisition of the velocity of the leading vehicle in case of communication delay or even failure, a random forest velocity prediction method is applied to eCACC based on the fusion of historical and sensor information, including the historical velocity and acceleration of leading vehicle as well as workshop spacing obtained from radar. On this basis, the velocity of the leading vehicle 1 can be accurately estimated in case of sudden communication failure. Random forest is a learning model integrated based on classification and regression trees (CARTs), which constructs multiple decision trees with randomly selected features and data samples and makes predictions using voting or averaging. In a random forest, the information entropy and information gain of a decision tree can be expressed as:

(22) $$\left\{\begin{array}{l} E=-\sum_{i=1}^{C}p_{i}\log_{2}(p_{i})\\ IG(D,A)=E(D)-\sum_{v=1}^{V}\frac{\left|D_{v}\right|}{\left|D\right|}E(D_{v}) \end{array}\right.$$

where C is the number of categories, and $p_{i}$ is the probability of category i. IG(D,A) is the information gain obtained by dividing the dataset D over the feature A, E(D) is the information entropy of the dataset D, v is the possible values of the feature A, and $D_{v}$ is the sub-dataset when the value of the feature A is v.

Thus, the prediction result of a random forest is:

(23) $$\hat{y}=\frac{1}{m}\sum_{i=1}^{m}y_{i}$$

where $\hat{y}$ is the prediction result of the random forest, $y_{i}$ is the prediction result of each decision tree, and m is the number of decision trees.

To solve the optimal control problem involving spacing error, velocity error, and economy error, the objective function can be written as:

(24) $$\begin{array}{l} J=\int_{0}^{t_{0}}[J_{S,i}(t)+J_{V,i}(t)+J_{Motor,i}(t)]dt\\ \left\{\begin{array}{l} J_{S,i}(t)=K_{1}{\left\|Err_{S,i}(a_{i}(t),t)\right\|}^{2}\\ J_{V,i}(t)=K_{2}{\left\|Err_{V,i}(a_{i}(t),t)\right\|}^{2}\\ J_{Motor,i}(t)=K_{3}{\left\|Err_{Motor,i}(a_{i}(t),t)\right\|}^{2} \end{array}\right. \end{array}$$

The corresponding Hamiltonian function can be expressed as:

(25) $$H(a_{i}(t),t)=K_{1}{\left\|Err_{S,i}(a_{i}(t),t)\right\|}^{2}+K_{2}{\left\|Err_{V,i}(a_{i}(t),t)\right\|}^{2}+K_{3}{\left\|Err_{Motor,i}(a_{i}(t),t)\right\|}^{2}$$

where $K_{1}$, $K_{2}$, and $K_{3}$ are weight coefficients.

Consequently, the optimal solution can be obtained by satisfying the minimum of the Hamiltonian function in a finite set under constraints, which can be written as:

(26) $$\begin{array}{l} \Delta a_{i}^{*}(t)=\arg\underset{\Delta a_{i}}{\min}[H(a_{i}(t),t)]\\ s.t.\left\{\begin{array}{l} S_{i}(t)-S_{i-1}(t)\geq L_{\min}\\ V_{\min}\leq V_{i}(t)\leq V_{\max}\\ a_{\min}\leq a_{i}(t)\leq a_{\max}\\ T_{Motor\min}\leq T_{Motor,i}(t)\leq T_{Motor\max}\\ n_{Motor\min}\leq n_{Motor,i}(t)\leq n_{Motor\max}\\ P_{Motor\min}\leq P_{Motor,i}(t)\leq P_{Motor\max} \end{array}\right. \end{array}$$

where $\Delta a_{i}^{*}$ is the optimal acceleration correction for vehicle i. $L_{\min}$ is the minimum spacing. $T_{Motor,i}$, $n_{Motor,i}$, and $P_{Motor,i}$, respectively, represent the torque, speed, and power of motor employed on vehicle i. min and max denote the minimum and maximum limits of the corresponding variables, respectively.

Further, the reference datasets that eCACC feeds back to the FCEV platoon and the bottom-level distributed controllers can be written as:

(27) $$\left\{\begin{array}{l} a_{i}^{*}(t)=a_{1}(t)+\Delta a_{i}^{*}(t)\\ P_{load,i}(t)=\gamma_{Motor}(t)\cdot \frac{m_{i}\cdot a_{i}(t)\cdot V_{i}(t)}{3600} \end{array}\right.$$

where $a_{i}^{*}$ is the demand acceleration for vehicle i. $P_{load,i}$ is the powertrain demand power sequence of vehicle i, containing the comprehensive optimization information of eCACC.

By comprehensively considering the spacing error, velocity error and economy error of the vehicle platoon, the dual optimization control of can be achieved, thus improving the car-following effectiveness and economic performance of the vehicle platoon. In addition, the generated reference datasets with comprehensive optimization information can be used in the bottom-level control to further explore the energy-saving potential.

### 3.3. EMS Based on ECMS in Bottom-Level Distributed Controllers

**Figure 7:**
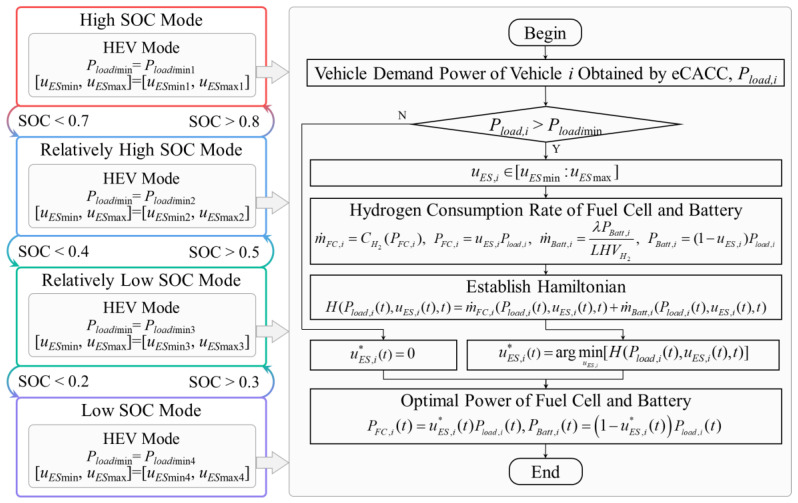
Implementation of EMS based on ECMS.

In order to reduce the energy consumption of power components and thereby further improve the economy of the FCEV platoon, optimal control for multiple energy sources is necessary. In the bottom-level distributed controllers for each FCEV individual, the proposed EMS optimizes the operation of multiple energy sources by analyzing reference datasets so as to further improve economy, and the implementation is shown in Figure 7.

In the framework of EMS, four control modes are categorized according to the battery SOC, including modes of high SOC, relatively high SOC, relatively low SOC, and low SOC. Since the special control of the power components is not required when the vehicle is braking, each control mode is only applicable in driving condition. In order to minimize the energy consumption of multiple power sources including the fuel cell and the battery, the objective function can be expressed as:

(28) $$\begin{array}{l} J=\int_{0}^{t_{0}}[J_{FC,i}(t)+J_{Batt,i}(t)]dt\\ \left\{\begin{array}{l} J_{FC,i}(t)={\dot{m}}_{FC,i}(P_{load,i}(t),u_{ES,i}(t),t)\\ J_{Batt,i}(t)={\dot{m}}_{Batt,i}(P_{load,i}(t),u_{ES,i}(t),t) \end{array}\right. \end{array}$$

where ${\dot{m}}_{FC}$ is the hydrogen consumption rate of the fuel cell. ${\dot{m}}_{Batt}$ is the equivalent hydrogen consumption rate of the battery. They can be determined by the following equation:

(29) $$\left\{\begin{array}{l} {\dot{m}}_{FC,i}(P_{load,i}(t),u_{ES,i}(t),t)=C_{H_{2}}(P_{load,i}(t)\cdot u_{ES,i}(t))\\ {\dot{m}}_{Batt,i}(P_{load,i}(t),u_{ES,i}(t),t)=\frac{\lambda \cdot \left(P_{load,i}(t)\cdot \left(1-u_{ES,i}(t)\right)\right)}{LHV_{H_{2}}} \end{array}\right.$$

where $P_{load}$ is the reference datasets generated by eCACC. $u_{ES}$ is the power distribution coefficient between the fuel cell and the battery. λ is the equivalent factor for converting the battery electricity consumption rate into the hydrogen consumption rate.

The corresponding Hamiltonian function can be written as:

(30) $$H(P_{load,i}(t),u_{ES,i}(t),t)={\dot{m}}_{FC,i}(P_{load,i}(t),u_{ES,i}(t),t)+{\dot{m}}_{Batt,i}(P_{load,i}(t),u_{ES,i}(t),t)$$

Therefore, the optimal solution can be gained by satisfying the minimum of the Hamiltonian function under constraints, which can be expressed as:

(31) $$\begin{array}{l} u_{ES,i}^{*}(t)=\arg\underset{u_{ES,i}}{\min}[H(P_{load,i}(t),u_{ES,i}(t),t)]\\ s.t.\left\{\begin{array}{l} u_{ES\min}\leq u_{ES,i}(t)\leq u_{ES\min}\\ P_{FC\min}\leq P_{FC,i}(t)\leq P_{FC\min}\\ P_{Batt\min}\leq P_{Batt,i}(t)\leq P_{Batt\min}\\ \Delta P_{FC\min}\leq \Delta P_{FC,i}(t)\leq \Delta P_{FC\min} \end{array}\right. \end{array}$$

where $u_{ES,i}^{*}$ is the optimal distribution coefficient for vehicle i. $P_{FC,i}$ and $P_{Batt,i}$, respectively, represent the fuel cell power and the battery power of vehicle i. $\Delta P_{FC,i}$ is the fuel cell power variation of vehicle i. min and max denote the minimum and maximum limits of the corresponding variables, respectively.

Under the optimal control input, the demand power of the fuel cell system and the battery can be written as follows:

(32) $$\left\{\begin{array}{l} P_{FC,i}(t)=P_{load,i}(t)\cdot u_{ES,i}^{*}(t)\\ P_{Batt,i}(t)=P_{load,i}(t)\cdot \left(1-u_{ES,i}^{*}(t)\right) \end{array}\right.$$

By combining reference datasets and sophisticated EMS in the bottom-level distributed controller, the operating states of the powertrain components are precisely optimized, thus contributing to a further improvement in economy of the FCEV platoon. 

Consequently, by integrating the eCACC in the top-level centralized controller and EMS in the bottom-level distributed controllers, eHCACC can improve the car-following performance of the FCEV platoon with the complete unleashing of the energy-saving potential.

## 4. Simulation and Discussion

**Figure 8:**
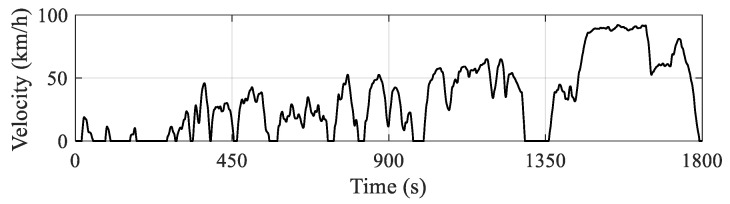
Curve of the CLTC driving cycle.

To verify the effectiveness of the proposed eHCACC, the comprehensive comparisons of car-following performance and economic performance are evaluated by a series of simulations. In terms of the evaluation index for validation, following errors and equivalent hydrogen consumption are employed for the evaluation. To ensure the authenticity of the validation, all driving conditions used for the leading vehicle in evaluations employ a standard driving cycle named China Light-duty Vehicle Test Cycle (CLTC), as shown in Figure 8. This standard driving cycle covers the common driving scenarios, including a low-speed segment representing urban driving, a medium-speed segment characterizing suburban driving, and a high-speed segment representing highway driving, thus ensuring the effectiveness and diversity of the comprehensive evaluation.

During the comparative validation process, a number of baselines are used. Specifically, methods for verifying economic car-following performance include:

- CACC: A vehicle platoon longitudinal control method considering spacing error and velocity error. In this study, the weighting coefficients $K_{1}$ and $K_{2}$ are set to 10 and 1, respectively.
- eCACC: A method of longitudinal control of a vehicle platoon comprehensively considering the spacing error, velocity error, and economy error, wherein the weighting coefficients $K_{1}$, $K_{2}$, and $K_{3}$ are set to 10, 1, and 4.23 × 10^−6^, respectively.

In addition, methods for verifying economic performance include:

- RB: A rule-based EMS competently distributing the power of the fuel cell and the battery [51]. Twelve fuel cell system demand power levels are determined by dividing the battery SOC into four levels: high SOC, relatively high SOC, relatively low SOC, and low SOC, as well as dividing the vehicle demand power into three levels: high, medium, and low. Note that key performance parameters of the fuel cell, including the maximum power, the efficient power, and idle power are set to 50, 20, and 2 kW, respectively.
- ECMS: A power distribution strategy for the fuel cell and the battery based on equivalent consumption minimization. Specifically, the operation of the fuel cell and the battery is categorized into four operating modes based on the battery SOC. And the optimal power distribution is solved by minimizing the equivalent hydrogen consumption. The equivalent factor λ is set to 2.48.

Consequently, eHCACC for the FCEV platoon is comprehensively evaluated in multiple scenarios. It is worth noticing that the simulations are conducted on MATLAB R2021b and performed on a personal computer equipped with an Intel i5-6300HQ processor and 8 GB of memory. Note that the time headway used in the simulation is 0.8 s and the time-delay constant is set to 0.5 s. Moreover, all simulations are conducted involving a fixed simulation step of 0.01 s.

### 4.1. Evaluation of Car-Following Performance

**Figure 9:**
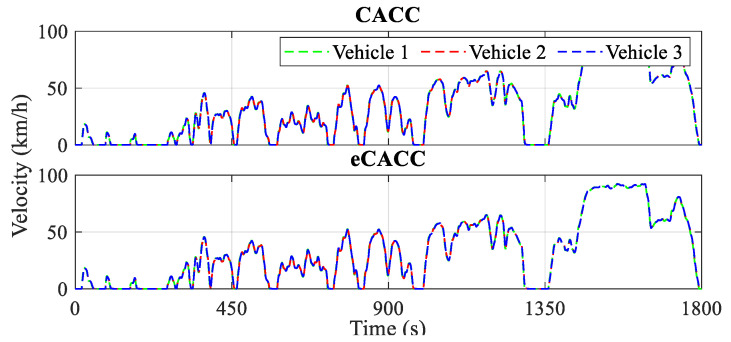
Curves of the vehicle velocity.

**Figure 10:**
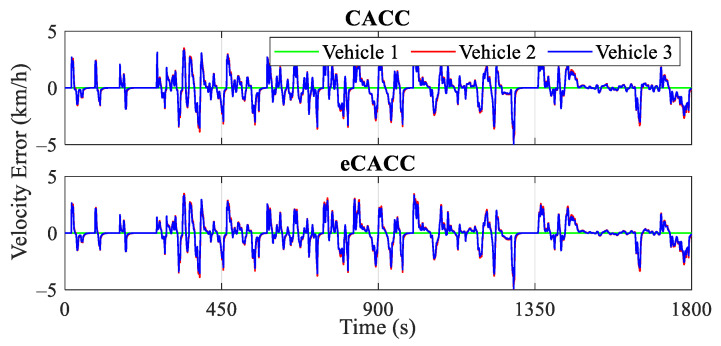
Curves of the velocity error.

**Figure 11:**
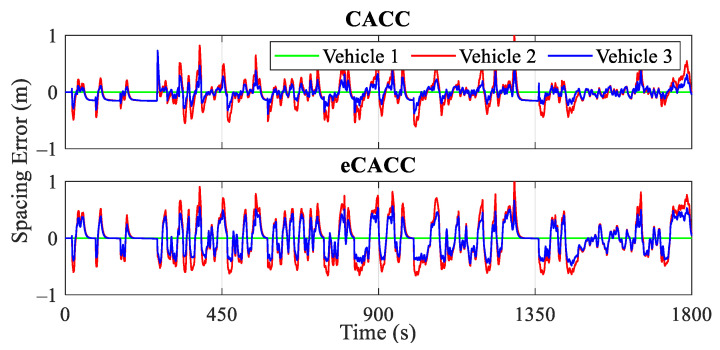
Curves of the spacing error.

The car-following performance of the top-level centralized controller directly affects the stability of the vehicle platoon, as well as the quality of the reference datasets provided to the bottom-level distributed controllers, and therefore, the validation of its effectiveness is a prerequisite. Figure 9, Figure 10 and Figure 11 present the evaluation in terms of vehicle velocity, velocity error, and spacing error of the vehicle platoon, respectively.

The actual velocity curves of all vehicles in the platoon under the CLTC driving cycle are shown in Figure 9. It can be seen that in CACC and eCACC strategies, the leading vehicle and following vehicles closely track the velocity curve for that standard driving cycle.

To ensure string stability, it is important to maintain a small velocity error and spacing error for the following vehicles and also ensure that the motion fluctuations of the leading vehicle are not amplified along the vehicle platoon. From the diagrams of velocity error and spacing error for vehicles 2 and 3, it can be observed that eCACC exhibits superior car-following performance throughout the CLTC driving cycle. Specifically, Figure 10 illustrates the velocity error curves of the vehicles in the platoon. It can be seen that both CACC and eCACC strategies for vehicles 2 and 3 exhibit stable velocity errors, with eCACC even slightly outperforming CACC, and the absolute value of errors is controlled to be below 5 km/h. Moreover, the magnitude of the vehicle velocity error along the platoon diminishes, in other words, the magnitude of the vehicle velocity error for vehicle 2 is smaller than that of vehicle 3. This indicates that the vehicle velocity error converges along the platoon, thereby ensuring the string stability. Similarly, this conclusion also applies to the spacing error. 

From the spacing error curves in Figure 11, it can be noted that with the involvement of the economic control, although the absolute value of the spacing error in eCACC is slightly larger than that in CACC (about a 0.1 m gap), both of the error absolute values are contained within 1 m. In addition, the spacing error also exhibits convergence along the platoon consistent with the velocity error, thus adequately ensuring the car-following performance and string stability of the vehicle platoon.

The comprehensive comparison verified the effectiveness of eHCACC in car-following performance and string stability.

### 4.2. Evaluation of Economic Performance

In addition to verifying the car-following performance, the superior control performance of eHCACC is also evaluated by considering economy, including energy efficiency and state changes for individual components.

#### 4.2.1. General Evaluation Results

**Figure 12:**
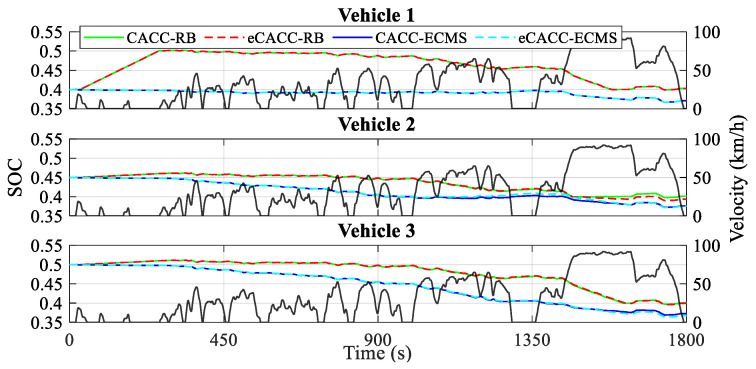
Curves of the battery SOC.

**Figure 13:**
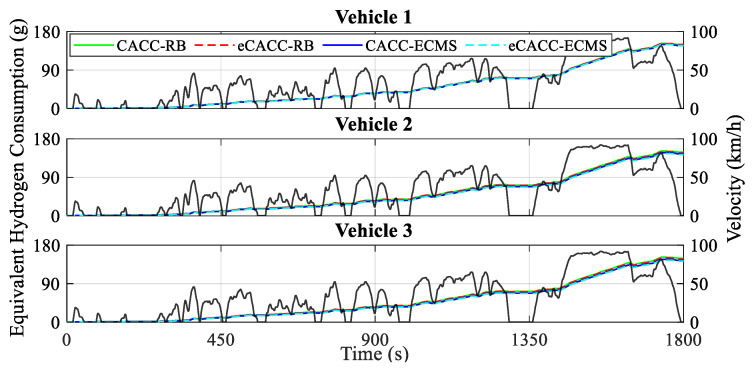
Curves of the equivalent hydrogen consumption.

In order to verify the economic performance in general, several static metrics for each vehicle obtained from the simulation are presented, including the battery SOC curves in Figure 12, the equivalent hydrogen consumption curves in Figure 13, and the economic comparison results in Table 2.

As shown in Figure 12 and Table 2, the initial SOC is different for each of the three vehicles; however, the different control methods in each vehicle result in different ending SOCs. Specifically, compared to RB, ECMS embodies a lower ending SOC, with a difference of almost 4% between them in vehicle 3. Meanwhile, such a difference in battery SOC leads to less energy consumption by ECMS, as shown in Figure 13. This is because taking on more of the power demand from the battery increases the chances of rationally regulating the operating states of the fuel cell, leading to significant economic payoffs. Moreover, with the eCACC strategy, the energy-saving efficiency of vehicles 2 and 3 is improved by about 1.5% compared with CACC, and the comprehensive optimality reaches almost 5% for vehicle 3, which fully verifies the effectiveness and strong impetus injected into the exploitation of energy-saving potential by combining centralized and distributed controllers.

#### 4.2.2. Energy-Saving Mechanism

**Figure 14:**
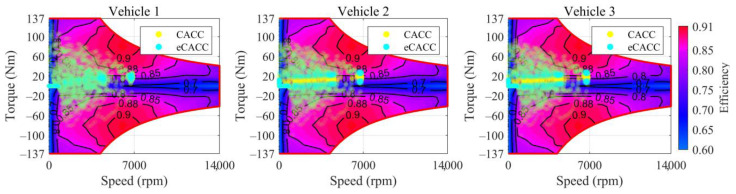
Distributions of the motor operating states.

**Figure 15:**
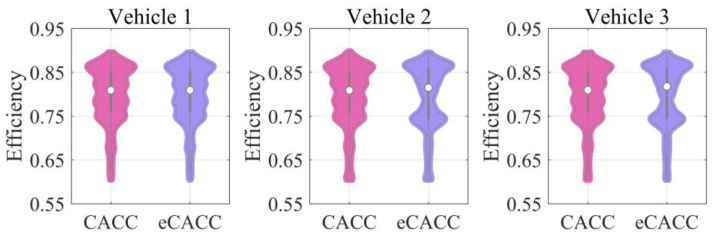
Frequency distributions of the motor operating states.

**Figure 16:**
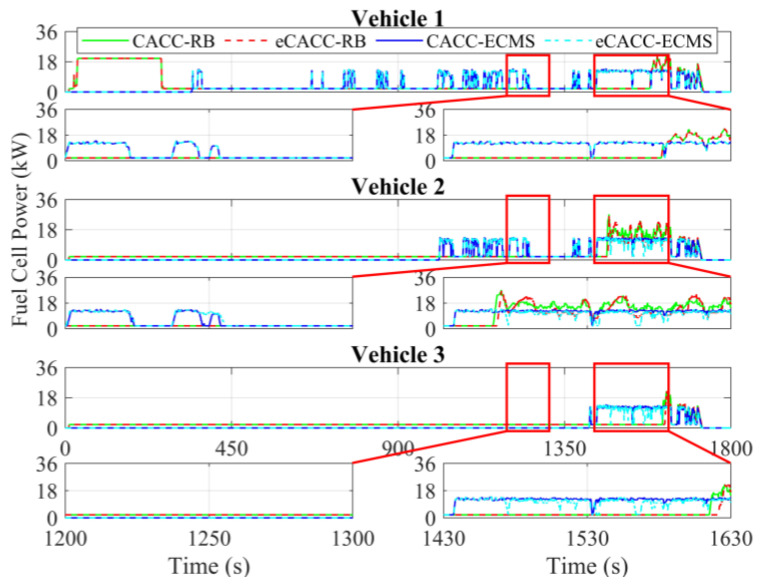
Curves of the fuel cell power.

**Figure 17:**
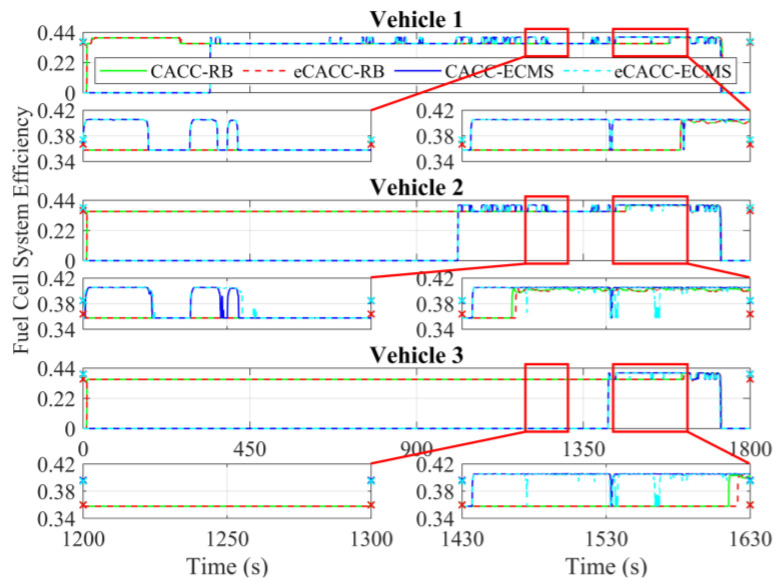
Curves of the fuel cell efficiency.

**Figure 18:**
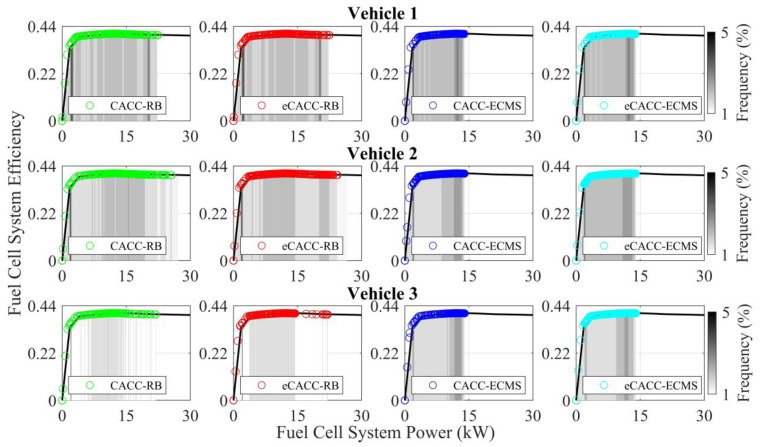
Distributions of the fuel cell efficiency.

**Figure 19:**
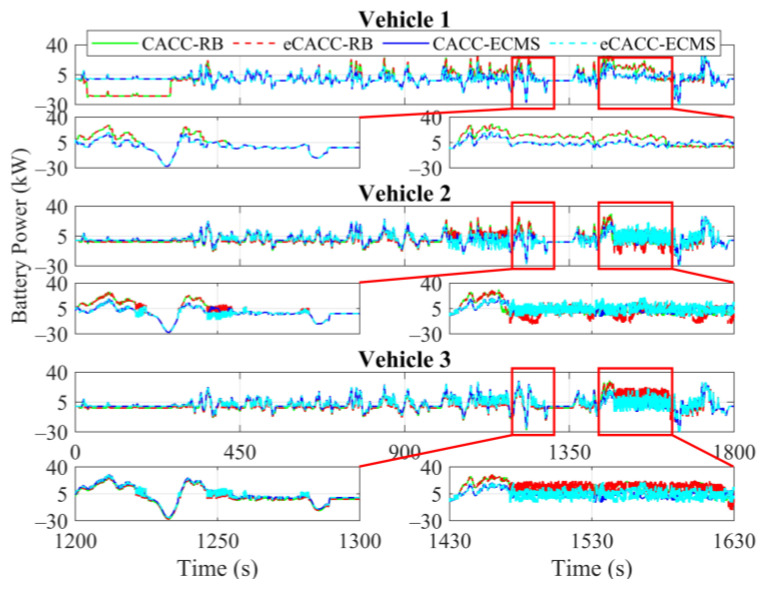
Curves of the battery power.

**Figure 20:**
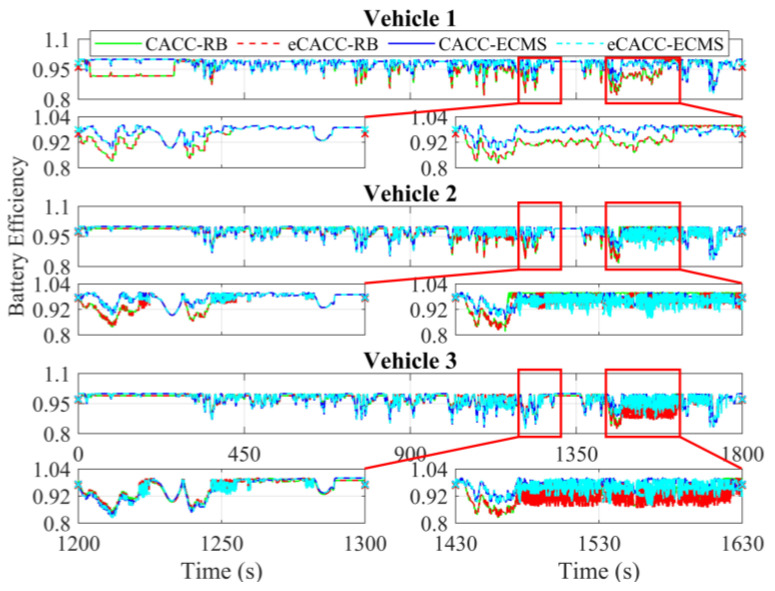
Curves of the battery efficiency.

In order to explore the energy-saving mechanism of eHCACC, the component operating states of individual vehicles are further analyzed, including the distributions of the motor operating states in Figure 14 and Figure 15, the diagrams of the fuel cell operating states in Figure 16, Figure 17 and Figure 18, and the diagrams of the battery operating states in Figure 19 and Figure 20. 

Figure 14 displays the distributions of the motor operating states. Note that the darker the color of the motor’s operating point in the diagram, the greater its frequency. It was found that compared to the leading vehicle, the following vehicles experience lower peak motor torque, which benefits from smoother longitudinal operation of the following vehicles under the car-following strategy. Meanwhile, for following vehicles, the motor in eCACC operates more in its high-efficiency region, especially when the motor torque is below 30 Nm. In order to more clearly express the distribution of the motor’s operating state, Figure 15 shows the frequency distribution of its operating efficiency. It can be noticed that the eCACC has the superior performance of transferring the operating state of the motor with an efficiency between 78% and 85% to an efficiency of 85% or more, thus exhibiting an average motor efficiency improvement of about 2%. As such, eCACC in the centralized controller can offer reference datasets with comprehensive optimization information for the distributed controllers, thus guaranteeing the economic improvement of eHCACC.

Figure 16 shows the fuel cell power curves under different methods. In the following vehicles, the ECMS-controlled fuel cell generally starts earlier than RB. Once the fuel cell starts, the ECMS enables it to work stably in the efficient power region (approximately 12 kW), which is especially noticeable in the simulation range of 1430 s to 1630 s in vehicle 2. Moreover, the fuel cell efficiency and its average are shown in Figure 17. It can be seen that for any individual vehicle, once the fuel cell has started and has been taken off idle power, ECMS always maintains the fuel cell efficiency at a high level of around 40.5%. In particular, the eCACC-ECMS achieves an average efficiency of about 41%. The reason for this phenomenon is that the ECMS can maximize the stable operation in the high-efficiency region while guaranteeing a full response to the demand power of powertrain, thus leading to a superior economic performance.

Figure 18 expresses the distribution of the fuel cell efficiency explicitly. It can be seen that the rear vehicles exhibit more concentrated fuel cell power distribution than the front vehicles. In addition, the fuel cell power under ECMS is more concentrated in the high-efficiency region, especially eCACC-ECMS in vehicle 2 and CACC-ECMS in vehicle 3, which delivers higher energy efficiency. The above simulation results fully validate the effectiveness of eHCACC in fuel cell energy-saving control.

Figure 19 and Figure 20 record the cell power and its efficiency. Since the battery is used to respond to the power gap between the powertrain and the fuel cell, the battery power exhibits a large fluctuation in vehicles 2 and 3, especially in the simulation range between 1430 s and 1630 s. Nevertheless, as can be seen in Figure 20, the battery carried by each vehicle applying eCACC-ECMS exhibits a slight average efficiency advantage of around 1% to 3%. This provides a positive contribution to the realization of the energy-saving advantages of the fuel cell under eHCACC, leading to optimized overall energy consumption and thus fully exploiting the energy-saving potential of the FCEV platoon.

Diverse perspectives suggest that the proposed eHCACC integrated by a top-level centralized controller and bottom-level distributed controllers performs excellently with impressive car-following stability and energy-saving performance for the cruising control of the FCEV platoon.

## 5. Conclusions

An eHCACC strategy is proposed for an FCEV platoon through a novel hybrid control architecture, enhancing energy-saving potential while ensuring stable car-following performance. Specifically, within the TCC of the hybrid control architecture, an eco-driving car-following method with economic optimization is proposed for eHCACC, improving following stability and economy in longitudinal dynamic and generating optimal reference datasets with comprehensive optimization information. In BDCs, superior energy management based on ECMS and comprehensive optimization information is employed, precisely achieving the control objectives of optimal following and economic performance for the FCEV platoon. Compared to the baselines, the equivalent fuel consumption of eHCACC can be reduced by approximately 5%, while maintaining approaching similar following performance, demonstrating state-of-the-art performance in both car-following stability and energy efficiency.

However, this study neglected to validate and evaluate the real-time performance of the control methodology. In future work, more effort will be invested in realizing hardware-in-the-loop and on-board controller real-time validation.

## Figures and Tables

**Table 1:** The detailed parameters of FCEVs in the vehicle platoon.

Parameters	Value	Unit
Mass	1860	kg
Tire Rolling Radius	0.35	m
Rolling Resistance Coefficient	0.015	/
Aerodynamic Drag Coefficient	0.3	/
Frontal Area	2	m^2^
Air Density	1.18	kg/m^3^
Motor Speed Range	0~14,000	rpm
Motor Torque Range	−137~137	Nm
Maximum Power of FC Stack	61.56	kW
Battery Capacity	40	Ah

**Table 2:** General comparison of economic performance by different methods.

Vehicle Individuals	Methods	Initial SOC	Ending SOC	EHC ^2^ per 100 km (kg/100 km)	EHC ^2^ (g)	HC ^1^ of the Fuel Cell (g)	EHC ^2^ of the Battery (g)	Optimality (%)
Vehicle 1	CACC-RB	0.400	0.403	0.906	149.693	159.564	−9.870	/
	eCACC-RB	0.400	0.403	0.906	149.693	159.564	−9.870	/
	CACC-ECMS	0.400	0.371	0.892	147.261	127.365	19.896	1.625
	eCACC-ECMS	0.400	0.371	0.892	147.261	127.365	19.896	1.625
Vehicle 2	CACC-RB	0.450	0.401	0.902	148.924	115.128	33.796	/
	eCACC-RB	0.450	0.394	0.891	147.077	107.759	39.318	1.547
	CACC-ECMS	0.450	0.376	0.877	144.802	92.400	52.402	2.768
	eCACC-ECMS	0.450	0.375	0.866	142.971	90.105	52.867	3.997
Vehicle 3	CACC-RB	0.500	0.401	0.906	149.493	79.026	70.467	/
	eCACC-RB	0.500	0.400	0.895	147.682	76.460	71.223	1.211
	CACC-ECMS	0.500	0.373	0.874	144.314	53.218	91.095	3.464
	eCACC-ECMS	0.500	0.368	0.864	142.594	47.635	94.959	4.614

^1^ HC represents hydrogen consumption. ^2^ EHC represents equivalent hydrogen consumption.

## Data Availability

The original contributions presented in the study are included in the article, further inquiries can be directed to the corresponding authors.
